# ProTarget: a Danish Nationwide Clinical Trial on Targeted Cancer Treatment based on genomic profiling – a national, phase 2, prospective, multi-drug, non-randomized, open-label basket trial

**DOI:** 10.1186/s12885-023-10632-9

**Published:** 2023-02-22

**Authors:** Tina Kringelbach, Martin Højgaard, Kristoffer Rohrberg, Iben Spanggaard, Britt Elmedal Laursen, Morten Ladekarl, Charlotte Aaquist Haslund, Laurine Harsløf, Laila Belcaid, Julie Gehl, Lise Søndergaard, Rikke Løvendahl Eefsen, Karin Holmskov Hansen, Annette Raskov Kodahl, Lars Henrik Jensen, Marianne Ingerslev Holt, Trine Heide Oellegaard, Christina Westmose Yde, Lise Barlebo Ahlborn, Ulrik Lassen

**Affiliations:** 1 Department of Oncology, Phase 1 Unit, Rigshospitalet, Blegdamsvej 9, Copenhagen, Denmark; 2 Department of Molecular Medicine/Department of Oncology, Aarhus University Hospital, and Institute of Biomedicine, Pharmacology/Precision Medicine, Aarhus University, Aarhus, Denmark; 3 Department of Oncology, Clinical Cancer Research Center, Aalborg University Hospital, Aalborg, Denmark; 4 Department of Clinical Oncology and Palliative Care, Zealand University Hospital, Roskilde, Denmark; 5 Department of Clinical Medicine, Faculty of Health and Medical Sciences, University of Copenhagen, Copenhagen, Denmark; 6 Department of Oncology, Herlev Gentofte Hospital, Herlev, Denmark; 7 Department of Oncology, Clinic of Precision Medicine, Odense University Hospital, Odense, Denmark; 8 Department of Clinical Research, University of Southern Denmark, Odense, Denmark; 9 Department of Oncology, Vejle Hospital and University Hospital of Southern Denmark, Vejle, Denmark; 10 Department of Clinical Genetics, Vejle Hospital and University Hospital of Southern Denmark, Vejle, Denmark; 11 Department of Oncology, Goedstrup Hospital, Goedstrup, and Department of Clinical Medicine, Aarhus University, Aarhus, Denmark; 12 Center for Genomic Medicine, Rigshospitalet, Copenhagen, Denmark

**Keywords:** Cancer genetics, Targeted therapies, Clinical trials, Cancer immunotherapy, Precision oncology, Tumor-agnostic therapy

## Abstract

**Background:**

An increasing number of trials indicate that treatment outcomes in cancer patients with metastatic disease are improved when targeted treatments are matched with druggable genomic alterations in individual patients (pts). An estimated 30–80% of advanced solid tumors harbor actionable genomic alterations. However, the efficacy of personalized cancer treatment is still scarcely investigated in larger, controlled trials due to the low frequency and heterogenous distribution of druggable alterations among different histologic tumor types. Therefore, the overall effect of targeted cancer treatment on clinical outcomes still needs investigation.

**Study design/methods:**

ProTarget is a national, non-randomized, multi-drug, open-label, pan-cancer phase 2 trial aiming to investigate the anti-tumor activity and toxicity of currently 13 commercially available, EMA-approved targeted therapies outside the labeled indication for treatment of advanced malignant diseases, harboring specific actionable genomic alterations. The trial involves the Danish National Molecular Tumor Board for confirmation of drug-variant matches. Key inclusion criteria include a) measurable disease (RECIST v.1.1), b) ECOG performance status 0–2, and c) an actionable genomic alteration matching one of the study drugs. Key exclusion criteria include a) cancer type within the EMA-approved label of the selected drug, and b) genomic alterations known to confer drug resistance. Initial drug dose, schedule and dose modifications are according to the EMA-approved label. The primary endpoint is objective response or stable disease at 16 weeks. Pts are assigned to cohorts defined by the selected drug, genomic alteration, and tumor histology type. Cohorts are monitored according to a Simon’s two-stage-based design. Response is assessed every 8 weeks for the first 24 weeks, then every 12 weeks. The trial is designed similar to the Dutch DRUP and the ASCO TAPUR trials and is a partner in the Nordic Precision Cancer Medicine Trial Network. In ProTarget, serial fresh tumor and liquid biopsies are mandatory and collected for extensive translational research including whole genome sequencing, array analysis, and RNA sequencing.

**Discussion:**

The ProTarget trial will identify new predictive biomarkers for targeted treatments and provide new data and essential insights in molecular pathways involved in e.g., resistance mechanisms and thereby potentially evolve and expand the personalized cancer treatment strategy.

Protocol version: 16, 09-MAY-2022.

**Trial registration:**

ClinicalTrials.gov Identifier: NCT04341181.

Secondary Identifying No: ML41742.

EudraCT No: 2019–004771-40.

## Background

Personalized cancer care is rapidly evolving, as the biological understanding of the individual’s cancer disease increases. A deeper understanding of disease and host at the genomic level, coupled with accessible and affordable multiplex analysis of the transcriptome, proteome, and other aspects of the cancer, is leading the way towards new paradigms in the treatment of cancer, relying not solely on the tissue of origin, but on molecular tumor profiling.

Although actionable molecular targets are frequent in cancers, the heterogenous distribution across tumor types makes traditional randomized phase 3 clinical trials in precision medicine rare, especially in less frequently encountered molecular targets. However, evidence is mounting through small clinical trials, case series, and case reports that patient outcomes are improved when a targeted treatment is matched to a tumor harboring the molecular target [1–4]. Estimates are that 30–80% of advanced solid tumors harbor actionable genomic alterations [5–8].

Several challenges exist for identifying and providing the relevant treatment to the patients at need. Many oncologists and pathologists have sparse access to comprehensive genomic profiling for screening purposes and experts for interpretation of genomic test reports to guide scientifically informed decisions about the optimal use of targeted agents [9]. The relevant drug will in many cases be an already marketed drug to be prescribed outside the labeled indication or an investigational drug accessible only in a clinical trial. Marketed drugs may not be available to the treating physician due to reimbursement issues for patients treated in lieu of health insurances, or the use of the drug may not be approved for use in publicly funded health care systems due to high costs and/or sparse scientific evidence in the tumor type treated. As access to drugs may be limited and data acquisition and reporting may be sporadic in patients treated with molecular matched therapies in the off-label setting, the overall knowledge of clinical outcomes in this setting is limited. Currently, there are more than 30 marketed drugs targeting molecular pathways frequently aberrant in human tumors, e.g., *EGFR*, *BRAF*, *MET* and *KIT,* with several more in development (list of abbreviations, Table 1).

**Table 1:** List of abbreviations

Abbreviation	Definition
BRAF	B-Raf proto-oncogene, serine/threonine kinase
CA-125	Cancer antigene 125
CI	Confidence interval
CR	Complete response
CTCAE	Common Terminology Criteria for Adverse Events
ctDNA	circulating tumor DNA
EGFR	Epidermal growth factor receptor
EMA	European Medicines Agency
ERBB2	erb-b2 receptor tyrosine kinase 2
FDA	Food and Drug Administration
FFPE	Formalin fixed paraffin embedded
GCIG	Gynecological Cancer InterGroup
GCP	Good Clinical Practice
GOF	Gain of function
IMP	Investigational medical product
IMWG	International Myeloma Working Group
IV	Intravenous
KIT	Mast/stem cell growth factor receptor Kitq
LVEF	Left ventricle ejection fraction
MET	Hepatocyte growth factor receptor
DN-MTB	Danish National Molecular Tumor Board
OS	Overall survival
PCWG3	Prostate Cancer Working Group 3
PD	Progressive disease
PFS	Progression free survival
PR	Partial response
PSA	Prostate specific antigene
RANO	Response Assessment in Neuro-oncology Criteria
RECIST	Response Evaluation Criteria in Solid Tumours
SD	Stable disease
TIA	Transient ischemic attack
TMB	Tumor Mutational Burden
WES	Whole exome sequencing
WGS	Whole genome sequencing

Matching molecular targets to relevant drugs may improve outcomes in cancer patients although large scale prospective, randomized, controlled trials are yet to be concluded. The randomized phase II trial SHIVA by Tourneau with matched molecular target treatment vs. physicians choice failed to demonstrate any significant different in progression free survival (PFS) between the two arms [10]. However, the meta-analysis by Schwaederle et al. of 570 phase II trials comparing patients receiving molecularly matched treatment to non-matched treatment demonstrated more favorable outcomes for patients receiving matched therapies [11]. Several individual, non-randomized phase 1 and phase 2 trials comparing molecularly matched treatment to non-matched treatment also demonstrated improved outcomes for the matched treatment groups. The Initiative for Molecular Profiling and Advanced Cancer Therapy (IMPACT) study (NCT00851032) by Tsimberidou et al. [7] analyzed 1144 patients of whom 40.2% had one or more genomic aberrations. Patients receiving a targeted therapy matched to a genomic aberration had median PFS and overall survival (OS) of 4.1 months and 10.2 months, respectively, compared to 2.4 and 8.2 months for non-matched patients. The MOSCATO-01 (Molecular Screening for Cancer Treatment Optimization, NCT01566019) [12] trial by Massard et al. included 1035 adult patients. An actionable molecular alteration was identified in 411 of 843 patients with a molecular profile and 199 patients were treated with a targeted therapy matched to a genomic alteration. The PFS from molecularly matched therapy was compared to the PFS for the most recent therapy on which the patient had disease progression (PFS2/PFS1 ratio). The PFS2/PFS1 ratio was > 1.3 in 33% of the patients (63/193). Objective responses were observed in 22 of 194 patients (11%; 95% CI, 7–17%), and median overall survival was 11.9 months (95% CI, 9.5–14.3 months).

The Danish study, CoPPO (Copenhagen Prospective Personalised Oncology) included 500 patients undergoing biopsy followed by whole exome sequencing (WES) and RNA sequencing. One hundred one patients (20%) received matched treatment based on either pathogenic variants or RNA expression levels of targets available in early clinical trials or off-label treatment. Objective response according to RECIST v1.1 was observed in 15 of 101 patients (0% complete response, 15% partial response), with a median PFS of 12 weeks (95% confidence interval, 9.9–14.4) [13].

The growing number of U.S. Food and Drug Administration (FDA) and/or European Medicines Agency (EMA) approved drugs for specific molecular targets in distinct histologies may lead to increased off-label use. Multiple prospective trials have recently been initiated to gather data on safety and outcomes, as well as extensive molecular data. These studies rely on extensive molecular profiling and decision making at molecular tumor boards [14] for matching patients with targeted therapies in a phase 2 open-label, prospective, non-randomized design. The American TAPUR (Testing the Use of FDA Approved Drugs That Target a Specific Abnormality in a Tumor Gene in People With Advanced Stage Cancer, NCT02693535) [15], the Dutch DRUP (Drug Rediscovery Protocol; NCT 02925234 [16], and Canadian CAPTUR (Canadian Profiling and Targeted Agent Utilization Trial NCT03297606) [17] are ongoing trials with purpose and design similar to the ProTarget trial (NCT04341181).

The primary aim of this trial is to investigate the anti-tumor activity and toxicity of commercially available, EMA-approved targeted therapies outside the labeled indications in the treatment of advanced malignant diseases harboring specific actionable genomic alterations. This protocol has been prepared according to the Standard Protocol Items: Recommendations for International Trials (SPIRIT) guidelines [18].

## Design and methods

### Study design

ProTarget is a Danish nationwide, interventional, multi-drug, open-label, pan-cancer, non-randomized, prospective phase 2 basket trial which aims to investigate the efficacy and safety of targeted anticancer drugs when used off-label in patients with a malignant disease harboring an actionable genomic alteration. Patients are recruited from eight different investigator sites at oncological centers. The trial aims to include 100 pts. annually.

**Fig. 1:**
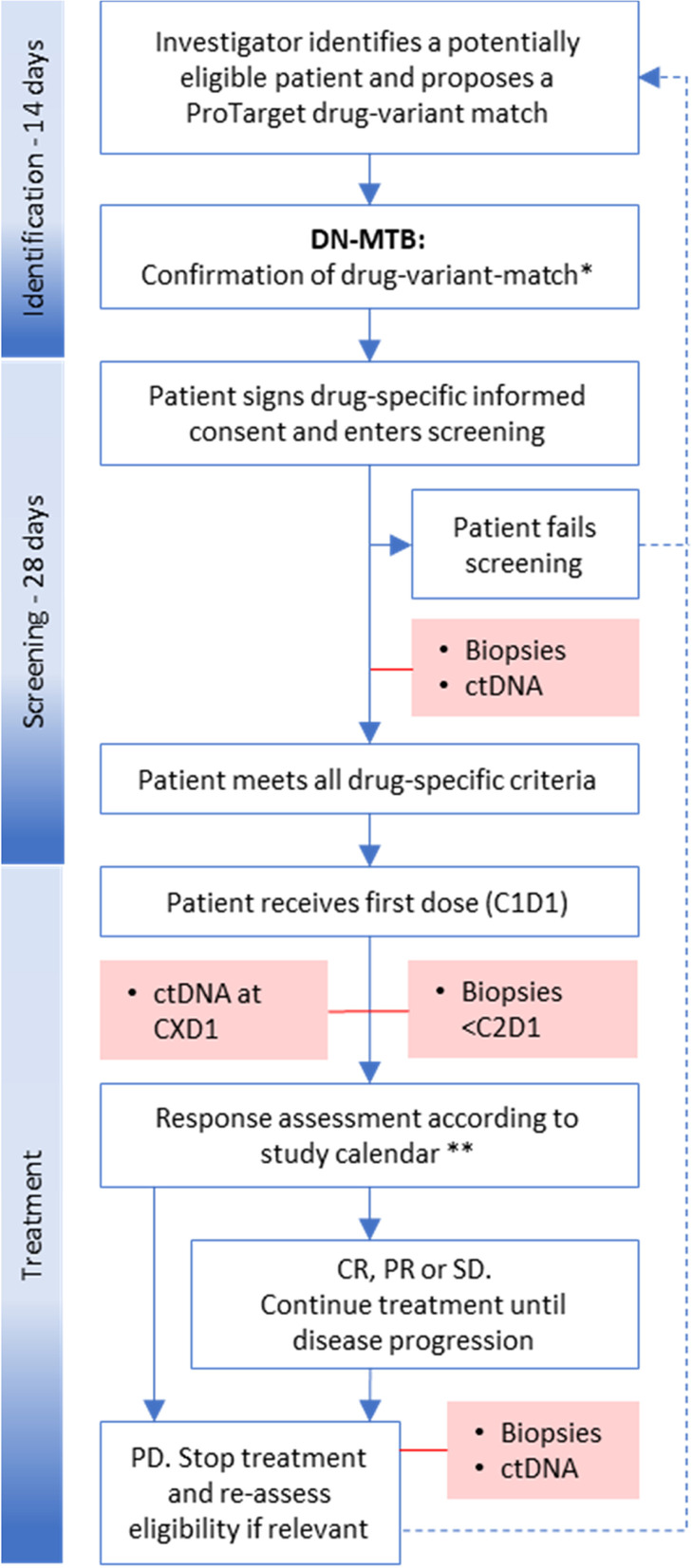
ProTarget Study Schema. Potentially eligible pts. are identified by local tumor genomic testing at each site. The investigator presents the genomic profile and clinical history and status of the patient and proposes the drug-variant match. If confirmed by the DN-MTB, the pt. can sign drug-specific informed consent and enter screening. Patients failing screening may be re-assessed for another drug if relevant. Patients meeting all eligibility criteria will start treatment. Fresh biopsies are taken at screening, on-treatment and at PD. Liquid biopsies are taken at screening and at CXD1 before dosing. Treatment is continued until PD or unmanageable toxicity. The pt. may be re-assessed for another drug if a matching alteration exists. *MTB may include as treatment options: A) Confirmation of ProTarget drug-variant-match, B) Treatment of an alternate ProTarget genomic alteration, C) Treatment of non-ProTarget variant on/off protocol or off-label, D) No treatment/protocol available. ** Every 8 weeks for 24 weeks, then every 12 weeks. Abbreviations: DN-MTB: Danish National Molecular Tumor Board, ctDNA: circulating tumor DNA, C1D1: cycle one day one, CXD1: any cycle day one, CR: complete response, PR: partial response, SD: stable disease, PD: progressive disease

Patients are identified through local genomic testing at each investigator site (Fig. 1). The proposed drug-variant-match (including full tumor genomic profile, tumor type and brief anonymous clinical case summary) must be submitted to and confirmed by the Danish National Molecular Tumor Board (DN-MTB) before informed consent can be obtained. The patient must meet all general and drug-specific criteria (Tables 2 and 3) before dosing in the trial. Fresh tumor biopsies are mandatory and obtained pre-treatment, during cycle one, and at progressive disease (PD). Breast or prostate cancer pts. with bone-only-disease, or patients with primary brain tumors, are eligible based on results from liquid biopsies only (e.g., circulating tumor DNA (ctDNA)). Liquid biopsies are collected from all patients at day 1 in each treatment cycle prior to dosing. Response assessment is performed every 8 weeks during the first 24 weeks, and then every 12 weeks.

**Table 2:** General inclusion criteria

General inclusion criteria (abbreviated)	
1) Patients ≥18 years of age with a histologically proven locally advanced or metastatic malignant disease for whom no standard treatment is available or indicated.	
2) Ability to understand and the willingness to sign a written informed consent/assent document.	
3) ECOG performance status 0–2.	
4) Acceptable organ function.	
5) Measurable or evaluable disease (e.g., RECIST v1.1 for solid tumors), defined as at least one lesion that can be accurately measured in at least one dimension. Patients who have assessable disease by physical or radiographic examination but do not meet these definitions of measurable disease are eligible and will be considered to have evaluable disease.	
6) Patients must have one of the actionable alterations listed in Table 4: ProTarget Drugs and Acceptable Molecular Alterations	
7) For oral IMPs, patients must be able to swallow and tolerate oral medication and must have no known malabsorption syndrome.	
8) Women of child-bearing potential and men must agree to use highly effective contraception.

**Table 3:** General exclusion criteria

General exclusion criteria (abbreviated)	
1) Ongoing toxicity > CTCAE grade 2. Patients with ongoing peripheral neuropathy of ≥ CTCAE grade 3.	
2) Prior treatment with the selected study drug.	
3) Genomic alterations known to confer drug resistance.	
4) Current treatment with other anti-cancer therapy (cytotoxic, biologic, radiation, or hormonal other than for replacement). Medications prescribed for supportive care that may potentially have an anti-cancer effect (e.g., megestrol acetate, bisphosphonates) or ongoing castration-intent therapy for prostate cancer, are accepted if they have been started ≥1 month prior to enrollment.	
5) Female patients who are pregnant or nursing. Male and female patients who refuse to practice highly effective contraception methods.	
6) Patients with known progressive brain metastases determined by serial imaging or declining neurologic function in the opinion of the treating physician. Patients with previously treated brain metastases must be clinically stable for at least 1 month after completion of treatment and off steroid treatment for one month prior to study enrollment.	
7) Patients with preexisting uncontrolled cardiac conditions, LVEF known to be < 40%, stroke (including TIA) or acute myocardial infarction within 4 months before the first dose.	
8) Patients with acute gastrointestinal bleeding within 1 month of start of treatment.	
9) Patients with any other clinically significant medical condition which, in the opinion of the treating physician, makes it undesirable for the patient to participate in the study e.g., active infection, significant uncontrolled hypertension, severe psychiatric illness situations, or anticipated or planned anti-cancer treatment or surgery.	
10) Patients who do not meet drug-specific eligibility requirements for the drug selected.	
11) Patients whose disease is not measurable or assessable by radiographic imaging or physical examination (e.g., elevated serum tumor marker only) with the exception of ovarian cancer (CA-125) and prostate cancer (PSA).	
12) Patients with known allergy/hypersensitivity to the study drug.	

Patient recruitment began 24-Aug-2020 and is ongoing. Last patient, last visit is undefined, and cohorts will open and reach completion successively depending on variant identification in individual pts.

### Study objectives

The primary objective of the study is to evaluate the anti-tumor activity and toxicity of commercially available, EMA-approved targeted anti-cancer drugs used off-label to treat patients with advanced malignant disease harboring a known or predicted targetable genomic alteration.

The secondary objectives are 1) To perform refined biomarker analyses (e.g., WGS) on serial fresh tumor samples and liquid biopsies, and 2) to study mechanisms of resistance using serial fresh tumor and liquid biopsies.

### Study endpoints

Primary study endpoints are:

- Anti-tumor activity, defined as objective response, at 16 weeks assessed by disease specific response criteria
- Stable disease at 16 weeks
- Treatment-related and serious adverse events

Secondary study endpoints are:

- Duration of response, progression-free survival and overall survival
- Duration of study treatment (time on drug)
- Percentage of screened patients treated based on their molecular tumor profile

Exploratory study endpoints:

- Description of concordance between genomic tumor profile of pre-treatment tumor biopsies and genomic tumor profile according to tumor profiling tests that were used to enroll patients
- Identification of patterns of resistance based on serial tumor biopsies and liquid biopsies

### Study population and eligibility criteria

Patients with metastatic or advanced malignant disease with exhausted treatment options, or for whom no standard treatment exist, are eligible. The tumor must harbor a potentially actionable genomic alteration targetable by a drug accessible in the ProTarget Protocol and must be a cancer histology outside the FDA/EMA-labelled indication. Patients with a genomic alteration known to confer resistance to a specific drug (such as solvent front or gatekeeper mutations or traits causing redundant signaling) are not eligible to receive that agent. Additional inclusion and exclusion criteria may apply to specific drugs or drug-tumor type-variant matches (Table 4). In these cases, drug-specific eligibility criteria must be met after general eligibility criteria have been met.

**Table 4:** ProTarget Drugs and Acceptable Molecular Alterations

Drug	Acceptable Genomic Alterations^ab^	Excluded Genomic Alterations^c^
Alectinib	*EML4-ALK* fusions or mutations, *ROS1* fusions	None
Atezolizumab	MSI high	None
	*POLE* mutations: R150X, P286R, P286H, S297F, Y298fs, F367S, V411L, L424V, P436R, V437M, S459F, R573L, E597K, R665W, L698fs, R762W, R793C, K1008N, T1052M, R1111Q, L1235I, V1368M, R1519C, P1547S, R1826W, R1879C, Y1889C, S1892N, A1967V, A2213V, A2243T	
	*POLD1* mutations: W79L, P112fs, A930fs, N247I, R352C, Q461H, S478N, A864T, E1105D	
	TMB ≥10 mut/mb	
Avelumab	MSI high	None
	*POLE* mutations: R150X, P286R, P286H, S297F, Y298fs, F367S, V411L, L424V, P436R, V437M, S459F, R573L, E597K, R665W, L698fs, R762W, R793C, K1008N, T1052M, R1111Q, L1235I, V1368M, R1519C, P1547S, R1826W, R1879C, Y1889C, S1892N, A1967V, A2213V, A2243T	
	*POLD1* mutations: W79L, P112fs, A930fs, N247I, R352C, Q461H, S478N, A864T, E1105D	
	TMB ≥10 mut/mb	
Axitinib	*VEGFR1 (FLT1)*, *VEGFR2 (KDR)*, *VEGFR3 (FLT-4)* GOF mutations, amplification, or overexpression	None
Erlotinib	*EGFR* exon 19 deletions in the region E746-E759 *EGFR* mutations: E709A/G/K, E884K, G719A/C/S, S768I, L858R, L861Q, L833V	Any of the following *EGFR* mutations: L747S, T790M, or T854A Exon 20 insertions
Niraparib	Germline or somatic *BRCA1/BRCA2* inactivating mutations *ATM/ATR* mutations or deletions HRD positive ^d^	None
Pemigatinib	Mutations in *PDGFRA*, *PDGFRB*, or *PCM1-JAK2* fusions. *FGF/FGFR* amplifications, mutation and fusions	None
Trastuzumab plus Pertuzumab	*ERBB2* amplification, overexpression, or mutations: G309A, G309E, S310F, D769H, D769Y, L755S, V777L, V842I, E321G, R896C *ERBB2* P780insertions *ERBB2* deletions in the region L755–T759	None
Trastuzumab emtansine	*ERBB2* amplification, or overexpression, or presence of any activating *ERBB2* mutations	None
Vemurafenib plus Cobimetinib	*BRAF* V600E/D/K/R mutations	Any mutations in *MAP 2 K1, MAP 2 K2, MEK1, MEK2, NRAS*
Vismodegib	*PTCH1* deletion or inactivating mutations	*SMO* mutations: D473G/H/Y, W535L *GLI2* amplification

Patients are eligible to receive one of the listed drugs if they have a non-indicated cancer harbouring a molecular alteration matching the drug

MSI: micro satellite instability; TMB: tumor mutational burden; GOF: gain of function; HRD: homologous recombination deficiency

^a^Source is FDA approved drug label, manufacturer data, [19–21]; Illumina Basespace Knowledge Network, QCI Precision Insight

^b^For any of the genes listed, alterations such as point mutations, insertions, deletions, translocations and amplifications or overexpression may be acceptable to match a drug to that gene. If a proposed drug-variant match is not accepted by the automated matching rules process, consider requesting case review by the Molecular Tumor Board

^c^Detection of any of the alterations in this column will exclude the patient from receiving the matched drug treatment as these alterations are associated with drug resistance

^d^HRD is evaluated from cytoscan HD (ThermoFisher) SNP array where an HRD score is calculated based on the sum of loss of heterozygosity (LOH), telomeric allelic imbalance (TAI) and large-scale transitions (LST) [22]. When WGS data are available, HRD status can be supported by mutational signatures from Cosmic [23] and CO-Regulation Database (CORD [24])

A patient must meet all of the following criteria to be eligible to participate in this study:

Potential participants who meet any of the following criteria will be excluded.

### Study procedures

Patients, who meet all eligibility criteria will be included. General study procedures are described in the following.

#### Actionable genomic alterations and drug selection

Potentially eligible patients must have at least one of the actionable genomic alterations (somatic or germline) listed in Table 4: ProTarget Drugs and Acceptable Molecular Alterations.

identified in their tumor and no variants conferring resistance to the relevant targeted anticancer therapy. The genomic alteration may be identified by any tumor genomic test or immunohistochemistry test performed on any type of tumor specimen (fresh frozen, RNAlater-preserved, formalin fixed paraffin embedded (FFPE)) or on ctDNA obtained from plasma (liquid biopsy) in a laboratory accredited by the competent local regulatory authority. Central confirmation of the actionable genomic alteration is performed retrospectively but is not a requisite for initiation of treatment. If more than one actionable genomic alteration is identified, the drug with the higher level of evidence supporting its use is preferred [19]. If several drugs with similar mode of action are available (i.e., PD-1 or PD-L1 inhibitors) randomization is performed by the trial coordinating team using Research Electronic Data Capture (REDCap v. 10.6.18, Vanderbilt University). Stratification factors include investigator site and performance status. REDCap is a secure, web-based software application building managing data for research studies [25, 26]. It is hosted at the Capital Region, Denmark.

#### Study drugs, treatment assignment and plan.

As of August 2022, 13 drugs are available in ProTarget and administered as monotherapy, unless otherwise indicated. Alectinib (Alecensa®), atezolizumab (Tecentriq®), erlotinib (Tarceva®), cobimetinib (Cotellic®) and vemurafenib (Zelboraf®) in combination, trastuzumab (Herceptin®) and pertuzumab (Perjeta®) in combination, trastuzumab emtansine (Kadcyla®), and vismodegib (Erivedge®) are supplied by Roche. Avelumab (Bavencio®) is supplied by Pfizer, as part of an alliance between Pfizer and Merck (CrossRef Funder ID: 10.13039/100009945), and axitinib (Inlyta®) is supplied by Pfizer. Niraparib (Zejula®) is supplied by GSK. Pemigatinib (Pemazyre®) is supplied by Incyte.

The study drugs are administered in cycles of 21–28 days. Patients are followed according to standard of care, unless otherwise specified in the drug-specific study manual. Initial drug dose and schedule, dose modifications, and management of treatment-related toxicities are performed according to the FDA and/or EMA approved label. All patients are followed for protocol-specified toxicity and efficacy outcomes including tumor response, progression-free survival and overall survival as well as duration of treatment. Treatment-related adverse events are graded according to Common Terminology Criteria for Adverse Events (CTCAE) version 5.0 and followed up until 1 month after the last administration of study drug. For all orally formulated drugs pts. complete dosing diaries and drug accountability is performed after each cycle. All pts. are treated free-of-charge, costs for transportation and accommodation are covered by the Danish healthcare system.

#### Response assessment and treatment duration

Response evaluation is performed every 8 weeks for the first 24 weeks and subsequently every 12 weeks until disease progression or treatment discontinuation. For patients with solid tumors other than glioblastoma response will be evaluated using the revised Response Evaluation Criteria in Solid Tumors (RECIST) guideline v 1.1 [27] and/or GCIG criteria [28] in case of CA125-based evaluation of patients with ovarian cancer and/or PCWG3 criteria for prostate cancer patients [29]. Bone-only breast cancer pts. will be evaluated using the MDA criteria [30, 31]. Being a non-randomized trial with objective response or non-progression as primary endpoint, confirmation of PR and CR is required ≥30 days after the first documentation of PR or CR, and documentation of non-progression per relevant diagnostic criteria for ≥16 weeks is required ≥2x and ≥ 28 days apart. For patients with multiple myeloma or B cell non-Hodgkin lymphoma, IMWG response criteria [32, 33] and CHESON/Lugano guidelines [34, 35] will be used, respectively. For glioblastoma patients, Response Assessment in Neuro-Oncology (RANO) criteria will be used [36]. Response is assessed by the local investigator. Study treatment will continue until unacceptable toxicity, PD, death, pregnancy, consent withdrawal or withdrawal at the discretion of the investigator. For patients treated with immunotherapy, treatment beyond radiographic progression is permitted provided the patient experiences clinical benefit, assessed by the local investigator.

#### Cohort definition and design

Each cohort is defined by the chosen study drug, relevant genomic alteration, and histologic tumor type (e.g., atezolizumab/TMB-high/prostate cancer). The ‘genomic alteration’ category is defined at gene level i.e., mutation, deletion, or amplification, e.g., *ERBB2-*mutation. Each cohort is monitored using a Simon-like two-stage ‘admissible’ monitoring plan to identify cohorts with evidence of activity [37, 38]. In short, eight participants are enrolled in stage one. If ≥1 patient achieves response on treatment (defined as ‘response’ per applicable criteria, or as stable disease for at least 16 weeks measured ≥2x and ≥ 28 days apart), an additional 16 participants are included in stage two, otherwise the cohort is permanently closed. If ≥5 out of 24 participants in stage two achieves response, further investigation of the drug-variant-tumor type combination is warranted. Response among ≤4 out of 24 participants will indicate lack of effect and the cohort is permanently closed. Patients are evaluable if they have received at least 1 cycle of oral drug or 2 administrations of IV drug, and if response is radiologically or clinically evaluable. Non-evaluable patients will be replaced.

### Clinical data

Data are captured on electronic Case Report Forms (eCRFs) using REDCap with capture of adverse events pr. CTCAE v. 5.0, vital signs and physical examination at the beginning of every treatment cycle and at end of treatment.

### Biological samples

Fresh tumor biopsies are preferably either 18G core-needle biopsies (3 samples, specimen length 22 mm) or surgical resection samples. Two samples are stored in RNA*later* (Life Technologies) to determine DNA aberrations and changes in RNA expression during treatment, and one sample is formalin-fixed and paraffin-embedded (FFPE) for histopathologic analyses (including standard biomarker analyses where applicable). 7 mL EDTA whole blood is collected during screening to determine background DNA variation and the presence of germline variants. Liquid biopsies (ctDNA) are collected in every cycle as peripheral blood in BCT tubes (Streck Laboratories, Omaha, NE, USA) as previously described [39]. Biopsies and blood samples will be stored in a research biobank for up to 5 years after the end of the ProTarget study and then destroyed.

### Sample analysis

Fresh tumor biopsies are analyzed by whole genome sequencing (WGS), RNA sequencing, and CNV-analysis (Illumina PCR-free, minimum 60x coverage, and Cytoscan) to identify genomic and phenotypic changes in the cancer cells during treatment as previously described [40, 41].

### Statistical considerations

Each cohort is monitored using a Simon-like two-stage ‘admissible’ monitoring plan. Admissible designs lie between MiniMax and Optimal designs and have good characteristics of both (i.e., small maximum sample size, and low expected sample size under the null hypothesis of low activity). A true response rate (defined as CR/PR or SD at 16 weeks) of less than 10% will be considered of no clinical interest. A response rate of 30%, although not comprehensively reflecting efficacy [42] or more will be considered of sufficient interest to warrant further study in a confirmatory trial, as outlined in DRUP and TAPUR trials. This monitoring plan has 85% power and an alpha error rate of 7.8%. These operating characteristics were selected to represent a reasonable compromise between high power, low false positive rates, and desire for small sample sizes, especially in stage one.

## Ethic considerations and dissemination

The study is conducted according to the international standards of ICH/Good Clinical Practice, monitored by the independent Danish GCP Units, and in full conformance with the “Declaration of Helsinki” and the Danish laws and regulations. The Protocol is approved by the Danish Ethics Committee (H-19089780; date of approval: 19-JUN-2020), the Danish Data Protection Agency (P-2020-210; 03-MAR-2020) and the Danish Medicines Agency (EudraCT 2019–004771-40; 17-FEB-2020).

All patients are informed about genetic findings revealed by genomic analysis and their potential consequences. In case of incidental findings with potential serious consequences for either the patient or the patient’s family, the patient will be offered referral for genetic counseling. Furthermore, the patients are informed that personal study-related data will be used by the Sponsor in accordance with the General Data Protection Regulation, the Data Protection Act and the Health Act. All patients are assigned a unique study ID to maintain patient confidentiality, if/when data is pooled with data from collaborating studies. In accordance with Danish law, research subjects are covered by Danish health care liability insurance.

Protocol amendments and modifications will be submitted for approval to the competent authorities and all relevant collaborators (e.g., sites, pharmacies, monitors, and funders) will be informed by the trial coordinating team. Results will be published in international and peer-reviewed scientific journals and presented at international conferences. Positive, negative as well as inconclusive results will be published. Designation of authorships will be based on the criteria of the Vancouver Convention (ICMJE).

Upon the completion of a cohort, individual and/or pooled cohort results will be published. General study results (such as overall submission, accrual, toxicity and efficacy analyses, as well as concordance between historic and pre-treatment genomic tumor profiles) will be published when appropriate. Individual case reports will only be published if clinically relevant. Publications will be prepared according to the Reporting Recommendations for Tumour Marker Prognostic Studies (REMARK [43]) and Standards for Reporting of Diagnostic Accuracy Studies (STARD [44]) guidelines.

## Study status

**Fig. 2:**
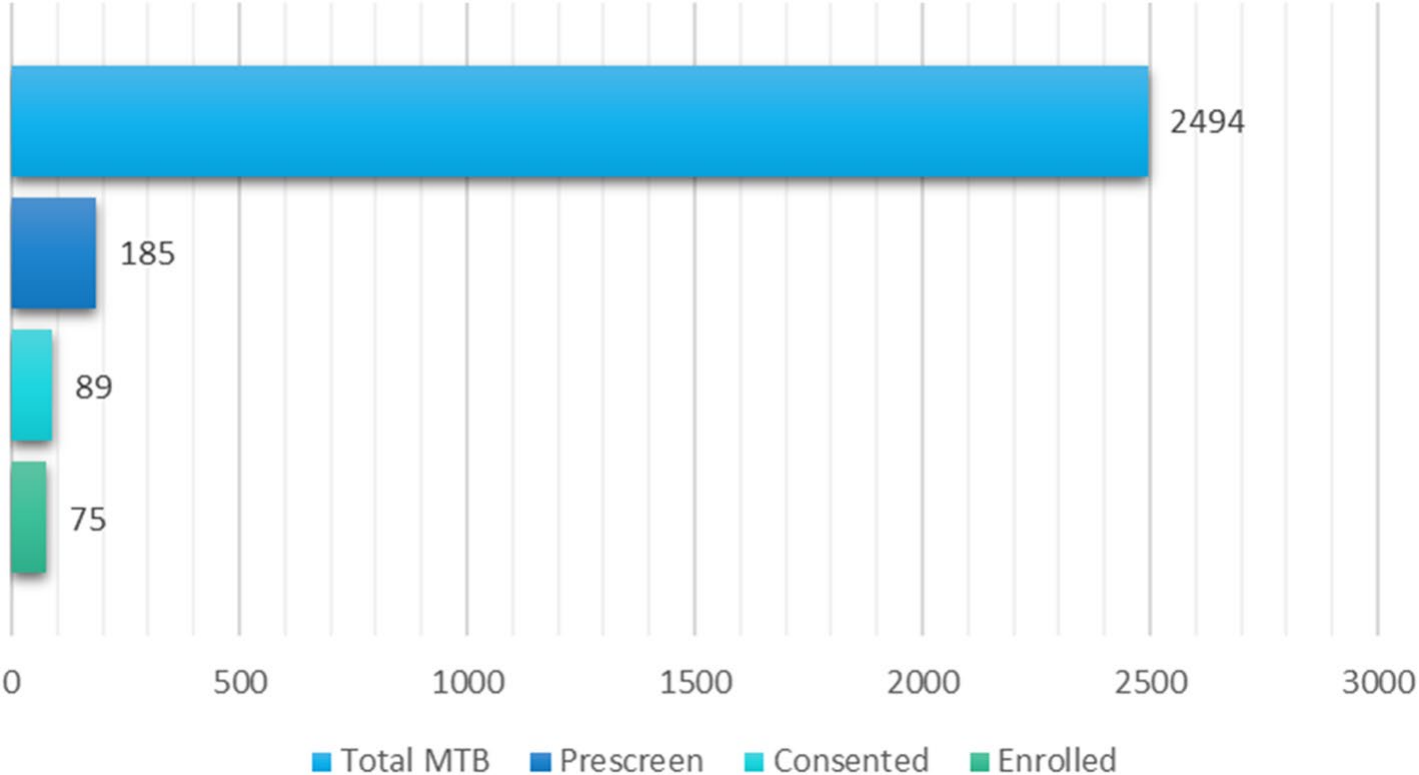
Patient enrollment status in ProTarget. The figure illustrates the number of molecular tumor profiles (Total MTB) assessed at the DN-MTB, and for ProTarget: the number of pre-screened pts. (Prescreen), number of pts. with informed consent (Consented), and number of pts. enrolled in treatment from study start 24-AUG-2020 to cut-off date 01-JUL-2022

From study start 24-AUG-2020 to cut-off date 01-JUL-2022, 185 pts. of 2.494 pts. evaluated at DN-MTB have been pre-screened and found potentially eligible for ProTarget (Fig. 2). Of these 185 potentially eligible pts., 89 pts. have signed informed consent to participate, 75 pts. have been enrolled in treatment while 14 pts. failed screening. Fifty-four cohorts have been opened.

## Collaboration

The present trial design will result in a number of cohorts consisting of rare combinations of genomic alterations and tumor types which will be difficult to complete. To accommodate this challenge and ensure that all cohorts will provide conclusive data, the protocol has been developed with a similar design as the DRUP (NCT02925234) and TAPUR (NCT02693535) trials and the Nordic Precision Cancer Medicine Trial Network [45] has been established. The Nordic Network is established between DRUP, and the Nordic trials: ProTarget (NCT04341181), IMPRESS-Norway (NCT04817956), MEGALiT (Sweden, NCT04185831) and FINPROVE (Finland, NCT05159245) with the aim of merging data for specific cohorts in common. The network is focusing on further aligning objectives, endpoints and eCRFs to facilitate data aggregation, which will be based on generally accepted principles and involve relevant pseudonymized data and clinical outcomes. Data sharing will comply with applicable legislation, ethical approvals as well as Data Sharing Agreements and scientific publications based on joint cohorts will be discussed for each cohort and coordinated by the Data Sharing Committee.

## Discussion and potential limitations

The ProTarget trial matches patients with non-curable malignant disease, harboring actionable genomic alterations, with relevant targeted drugs. The trial aims to evaluate the efficacy and safety of currently 13 EMA-approved targeted drugs, and study mechanisms of resistance using paired and serial tissue and liquid biopsies.

The ProTarget trial builds on the experiences and designs from matched therapy trials like DRUP and TAPUR and will provide data for the growing network of similar trials. Unique for the ProTarget trial is the extensive genomic profiling provided by the repeat biopsy design. By analyzing the extensive molecular data pre-, on-, and post-treatment, new insights can be gained in molecular pathways involved in e.g., intrinsic and acquired resistance to targeted anti-cancer therapies, clonal evolution, and predictive factors.

The DN-MTB plays a pivotal role in ProTarget; this national, multidisciplinary collaboration is attended by oncologists, molecular biologists, bioinformaticians, pathologists, and clinical geneticists from eight centers across Denmark covering 5.7 million inhabitants. It provides an opportunity for multidisciplinary evaluation and discussion of each case with regards to actionable genomic alterations, strong (dominant) onco-drivers, and potential resistance mutations, combined with the clinical history, histopathology, and patient status. The DN-MTB reviews approximately 1200 genomic profiles annually, mainly WGS/WES and large NGS panels. Thus, the DN-MTB ensures thorough and multidisciplinary pre-screening of each candidate before inclusion in the trial.

Patients are identified by local testing by any method in any type of tissue or blood sample for rapid, broad pre-screening of potential candidates. However, if data are derived from small NGS panels or IHC testing, treatment decisions may be made on potentially incomplete data. Furthermore, the tumor may have developed new oncogenic drivers or resistance mechanisms after the initial testing. To address these issues, fresh tumor biopsies are taken at baseline, analyzed by WGS, and presented at the DN-MTB to ensure that the genomic alteration is still present and relevant for targeted treatment. Treatment initiation and continuation decisions are not per se dependent on these protocol specific tests but may help guide treatment decisions for the individual patient and improve understanding of drug efficacy.

Each cohort is defined by study drug, the actionable genomic alteration, and the histologic tumor type; a design that eventually will give rise to cohorts of rare variant/tumor type-combinations difficult to accrue the required initial 8 subjects. To accommodate this issue, the protocol has been designed in similar to the DRUP and TAPUR trials with a European data sharing agreement for sharing such cohorts.

Tissue and liquid biopsies are collected for translational research for deeper understanding of targeted therapy resistance when applying large scale molecular profiling. The large amount of molecular data combined with the prospective clinical data will enable future research projects in a variety of fields including providing data for inductive hypothesis generation. Liquid biopsies are still in development as a tool for monitoring tumor progression, treatment response and in screening for therapeutic resistance in the individual patient. By collecting paired and serial tissue and liquid biopsies, this trial aims to investigate this modality further and combine findings with the solid tissue WGS and prospectively acquired clinical data. Monitoring of mean variant allele frequencies of selected variants across tumor types during treatment as a surrogate for PFS or response rate is currently being investigated in early clinical trials [46]. Large scale prospective studies comparing ctDNA and tumor tissue DNA before and during targeted treatment are few [47] but hold potential for using ctDNA for diagnostic and prognostic purposes.

In conclusion, data from this trial can potentially identify new matches between targeted treatments and actionable tumor genomic alterations, provide evidence for non-efficacy for other tumor type/genomic alterations and identify potential safety issues in marketed targeted therapies. New insights can be provided using whole genome data for exploratory analysis of determinants of efficacy and resistance.

## Data Availability

Not applicable.
